# Low Inducer Concentrations at 10°C Promotes Soluble Recombinant Expression of Aedes aegypti Mosquito Midgut Proteases in E. coli

**DOI:** 10.21203/rs.3.rs-8217026/v1

**Published:** 2025-12-11

**Authors:** Daniel Fong, Neomi Millan, My Anh Le, Elizabeth Moreno-Galvez, Kevin Derisier, Abigail G. Ramirez, Kaelyn Pluta, Kimberly Houghton, Alberto A. Rascón

**Affiliations:** Arizona State University; San Jose State University; Arizona State University; San Jose State University; San Jose State University; Arizona State University; Arizona State University; San Jose State University; Arizona State University

**Keywords:** Aedes aegypti, Midgut, Proteases, Escherichia coli, Recombinant protein, Soluble expression, IPTG, Betaine, Disulfide bond

## Abstract

**Background:**

Soluble recombinant expression of *Aedes aegypti* mosquito midgut proteases in *Escherichia coli* prove to be difficult. These enzymes depend on disulfide bond formation for structural stability. Initial attempts in BL21(DE3) were unsuccessful due to a reducing cytoplasm. The use of T7 SHuffle cells (with a more oxidizing cytoplasm) led to soluble expression. However, other factors had to be altered (use of richer media and lower (> 25°C) growth temperature). Not all mosquito proteases were equally soluble. Therefore, given the importance of IPTG in initiating transcription and translation, we set out to determine if low IPTG concentrations (^3^ 0.1 mM) at 10°C would increase soluble production of midgut proteases. Additionally, we investigated the effect of the small molecule osmolyte betaine on the soluble expression of midgut proteases.

**Results:**

For this study, the focus was on *Aedes aegypti* Late Trypsin (AaLT), Early Trypsin (AaET), Serine Protease I (AaSPI), Serine protease V (AaSPV), and Juvenile Hormone Associated 15 (JHA15). The colder bacterial growth, along with low IPTG, slows the rate of transcription/translation of T7 RNA polymerase. Lower expression of T7 RNA polymerase, along with slower transcription activity at 10°C, prevents rapid simultaneous translation of midgut proteases. This allows recombinant proteases to properly fold. In addition, we found that different growth periods also varied among the proteases. Soluble expression for AaLT and AaET was maximal at 52 h post-induction, 72 h for JHA15, and 168 h for AaSPI and AaSPV. Surprisingly, for AaET, temperature was the only important factor. The addition of betaine to the growths had a more pronounced effect at higher (> 0.05 mM) IPTG.

**Conclusions:**

Low IPTG at 10°C slows the rate of transcription/translation of recombinantly expressed mosquito proteases in bacteria. By preventing rapid accumulation in the cell, prevents aggregation, and ultimately inclusion body formation. Betaine works better at higher IPTG concentrations, but more studies are needed to better understand how this osmolyte stabilizes proteins during recombinant bacterial expression. Nonetheless, this study provides a blueprint for researchers who have never attempted IPTG concentrations > 0.1 mM to recombinantly express proteins in bacteria.

## Background

Recombinant protein expression utilizing *Escherichia coli* is the most common and popular prokaryotic host due to several important factors. These include but are not limited to fast growth rates to achieve high cell densities, ease of well-developed molecular manipulation tools, cost effectiveness using inexpensive rich media cultivation methods, and more importantly, yielding an abundance of recombinant protein than can normally be produced from natural systems [1–4]. However, the rapid simultaneous transcription and translation of heterologous genes in *E. coli* often results in unfolded or misfolded proteins leading to protein aggregation and formation of inclusion bodies, especially in proteins that require some sort of post-translation modification (*e.g*., disulfide bond formation) [3–6]. Over the years, several review articles have touched on the use of different bacterial strains and growth cultivation conditions that minimize improper folding of recombinant heterologous proteins, especially those from eukaryotic organisms. Many strategies have been described, including the use of rich auto-inducing media, reduced growth temperatures, optimized codon usage, modifications of ribosome binding site sequences, the use of fusion tags, and the use of bacterial strains that have a more oxidizing cytoplasm or strains that support growth and folding at temperatures lower than 10°C (for extensive reviews, see [4–12]). Our own research focusing on the recombinant expression of *Aedes aegypti* mosquito midgut proteases have benefitted from many of the suggestions offered in these reviews [2, 13, 14].

The *Ae. aegypti* mosquito is a carrier of several potentially deadly viruses, such as Dengue (DENV), Zika (ZIKV), and Chikungunya (CHIKV) that may be transmitted to human hosts. Infection with any of these viruses may lead to minor symptoms closely matching the flu, but in severe cases can lead to hemorrhagic stages and death (DENV), microcephaly and Guillain-Barré syndrome (ZIKV), and severe joint and muscle pain (CHIKV) [15–17]. Unfortunately, due to climate change, rising temperatures and humidity, the presence and survival of the mosquito have increased affecting viral replication and viral transmission efficacy [18]. In fact, a global outbreak was observed in 2023 with DENV transmission and Dengue fever incidence increasing to over 6.5 million and 7,000 Dengue-related deaths in more than 80 countries [19, 20]. In the Southern United States, the presence of all three viruses has been observed with the potential of these viruses to be locally transmitted and infections to become permanently locally acquired [17, 21]. Fortunately, vector control utilizing insecticides and larvicides remain an effective strategy in minimizing viral pathogen transmission, but heavy reliance on pesticides is leading to resistance in *Ae. aegypti* [22, 23]. Other biological methods, such as the release of sterile males or *Wolbachia*-infected mosquitoes have been promising in reducing the mosquito population, but release acceptance and logistical issues have prevented their use [24, 25]. Given the importance of the mosquito in viral transmission other vector control strategies are needed.

Our research has focused on targeting the blood meal digestion process in the *Ae. aegypti* mosquito as a new vector control strategy. The female mosquito relies on a blood meal to acquire the nutrients and energy needed for egg production. To date, only 11 midgut proteases have been discovered to be expressed in response to a blood meal in the *Ae. aegypti* mosquito, with only six midgut proteases being fully or partially characterized *in vitro* [2, 13, 14, 26, 27]. The expressed midgut proteases are essential for the digestion and processing of blood meal nutrients into polypeptides and amino acids needed for egg production. Previous work from our lab has shown that certain midgut proteases directly affect not only blood meal protein digestion but have a direct effect on fecundity [28]. There is difficulty in studying and fully characterizing these proteases *in vitro* due to the limited amount of protease that can be isolated from a single mosquito. This means that thousands of mosquitoes must be blood fed, then processed for isolation from the complex mixture of unwanted proteins produced by the mosquito, as well as separated from the blood meal that contains its own complex set of proteins that need to be excluded to obtain a homogenous midgut protease sample. The task is daunting, especially since most of the midgut proteases have the same chemical and structural characteristics that would prevent purification using traditional methods (*e.g*., ion exchange, size exclusion, and even precipitation methods) [2, 26]. However, utilizing recombinant techniques, our lab was able to produce soluble and active mosquito proteases utilizing *E. coli* as a host [2, 13, 14, 26].

Initial midgut protease expression studies utilizing BL21(DE3) cells led to protein aggregation and inclusion bodies, which were then used to isolate and purify using a denaturation/renaturation folding scheme of four active midgut proteases [26]. However, the amount of protease isolated from the approach was not enough to help fully study the proteases *in vitro*. It is important to note, that the overarching goal of this work is to determine the kinetic parameters, substrate specificity, and protease structure to help biochemically and biologically determine the role of these enzymes in the blood meal protein digestion process. Those proteases that indeed have a direct effect on blood meal protein digestion and fecundity will be targeted for the development of protease inhibitors, leading to small molecule structure-based inhibitor design, and potentially a new vector control strategy. To achieve this, more recombinant proteases are needed. In 2018, the use of a bacterial strain with a more oxidizing cytoplasm (T7 SHuffle cells, NEB) proved to be essential when working with *Ae. aegypti* mosquito midgut proteases [2]. Most mosquito midgut proteases contain anywhere between 2–4 disulfide bonds that are essential for folding, structural stability, and activity [2, 13, 14, 26]. In BL21(DE3) strains and its derivatives, the site of recombinant protein expression is in the cytoplasm which has a reducing environment that prevents disulfide bond formation [3, 29, 30]. Although T7 Cells also express recombinant proteins in the cytoplasm, the cytoplasm is more oxidizing due to mutations in the thioredoxin reductase and glutathione reductase enzymes. These enzymes maintain a reducing cytoplasm in wildtype *E. coli*. Additionally, the use of richer media (Terrific Broth, TB) and lowering the bacterial growth temperature between 10° and 25°C helped with proper folding of mosquito proteases. However, the abundance and soluble expression of one specific protease, *Ae. aegypti* Late Trypsin (AaLT) was not as visible via SDS-PAGE analysis compared to the other two proteases isolated, serine protease VI (AaSPVI) and serine protease VII (AaSPVII). In addition, the abundance and soluble expression issues were also observed with early trypsin (AaET) [2]. The protease also autoactivated during recombinant expression making it difficult to purify.

Overall, changing the bacterial strain, use of richer media, and lowering the induction temperature [2] led to the improvement of four *Ae. aegypti* midgut proteases compared to the denaturation/renaturation scheme from 2011 [26]. Despite the visual low abundance of AaLT and AaET, the approach did lead to some properly folded and soluble active proteases [2]. However, a constant that was observed and not modified in both studies was the concentration of the inducer, *isopropyl-*β*-D-thiogalactopyranoside* (IPTG) which was held at 0.1 mM IPTG for all growths. To the best our knowledge, the lowest concentration reported in the literature to induce the production of soluble recombinant protein is 0.01 mM (10 mM) [31]. The authors indicated that inducing with higher concentrations of IPTG led to an increased amount of recombinant protein but mainly in its insoluble form. Whereas reducing the IPTG concentration led to more soluble protein. Therefore, given the importance of IPTG in initiating transcription and translation, for this study we set out to determine if lowering the IPTG concentration (at a constant lower 10°C temperature) would increase the abundance and promote better folding of AaLT and AaET in *E. coli*. Additionally, we investigated if the small molecule osmolyte betaine (which is thought to act as a molecular chaperone during *E. coli* recombinant expression [32]) would have an influence on the soluble expression of both AaLT and AaET. Results indicate that lowering the IPTG concentration does improve overall protein expression and solubility, while betaine in certain cases seem to affect production of soluble proteases in bacteria. As a proof of principle, we also report conditions that have led to the soluble production of proteases that have never been isolated: Serine Protease I (AaSPI) and Serine protease V (AaSPV); as well as a previously studied protease Juvenile Hormone Associated 15 (JHA15) that was difficult to express by our lab using BL21(DE3) cells.

## Methods

### Engineering and cloning of recombinant mosquito protease constructs

All recombinant midgut proteases were his_6_-tagged to facilitate visualization of the proteases using the InVision His-Tag staining system (Invitrogen, cat. #LC6030) during SDS-PAGE analysis. The AaLT, AaET, AaSPI, and AaSPV genes were all cloned into the pET28a vector (Novagen Cat. #69864–3) to yield proteases with an N-terminal his_6_-tag, while JHA15 was cloned into the pET29b vector (Novagen Cat. #69872–3) to yield a protease with a C-terminal his_6_-tag. More specifically, the AaLT zymogen form of the gene without the signal (leader) peptide sequence was cloned into the pET28a vector, as described (2) (Nguyen et al 2018). Similarly, the AaSPI gene without the signal sequence was cloned into the pET28a vector using the NdeI and HindIII restriction site sequences. The AaSPI primers (Table 1) and purified *Ae. aegypti* cDNA (as prepared in Rascon et al 2011) were co-incubated with the GoTaq Green Master Mix (Promega, cat. #M7122, Madison, WI) and amplified following the manufacturer’s protocol but with an annealing temperature of 62°C (20 sec). As for AaET, the gene was cloned with a heterologous enterokinase cleavage site into the pET28a vector to help minimize auto-activation of the protease during bacterial expression, yielding the N-terminally his_6_-tagged protease, as described (26) (Rascon et al 2011). Likewise, AaSPV was similarly cloned with a heterologous enterokinase cleavage site into the pET28a vector using the NdeI and XhoI restriction site sequences. AaSPV primers (Table 1) and purified *Ae. aegypti* cDNA were co-incubated with the GoTaq Green Master Mix and amplified following the manufacturer’s protocol but with an annealing temperature of 60°C (20 sec). Lastly, the JHA15 gene was similarly cloned with the heterologous enterokinase cleavage site into the pET292b vector using the NdeI and XhoI restriction sites (without a stop codon to ensure the addition of the C-term his_6_-tag). JHA15 primers (Table 1) and purified *Ae. aegypti* cDNA were co-incubated with the GoTaq Green Master Mix and amplified following the manufacturer’s protocol but with an annealing temperature of 60°C (20 sec). Once all plasmid constructs were engineered, DNA was sent to ELIM Biopharmaceuticals, Inc. (Hayward, CA) for DNA sequencing and sequenced verified using NCBI BLAST.

### Recombinant protease expression in T7 SHuffle *E. coli* cells

Small scale (50 mL) growth experiments were set to test the different low IPTG concentrations in the presence or absence of betaine. Plasmid constructs encoding the proteases of interest (25 to 50 ng pDNA) were transformed into aliquots of 25 mL SHuffle T7 Express Competent *E*. *coli* Cells (New England Biolabs #C3029J) and plated onto LB agar kanamycin (30 μg/mL) plates. Plates were then incubated overnight at 30°C for 16–18 h. The next day, single colonies were selected and sub-cultured in LB media supplemented with 30 μg/mL kanamycin and grown in a temperature-controlled shaker set at 30°C for 16–18 h (220 rpm). The optical density at 600 nm (OD_600_) of the overnight cultures were determined using a Nanophotometer NP80 (Implen) and used to start the 50 mL growths at an OD_600_ of ~0.05. All growths were grown in 50 mL terrific broth (TB) media (Fisher Bioreagents, cat. #BP2468–2) supplemented with glycerol using sterile 125 mL Erlenmeyer flasks (2) (Nguyen et al 2018).

For AaLT and AaET growth experiments, a total of 100 replicate flasks for each was prepared, each corresponding to 10 flasks of the different IPTG concentrations tested (0.1, 0.05, 0.025, 0.0125, and 0.00625 mM) in the presence or absence of 1 mM betaine (Thermo Scientific, cat. #164550010). All growths were initially set at 30°C (220 rpm) in a temperature-controlled shaker until the OD_600_ of each reached between 0.5 and 0.7. At this stage, the temperature of the shaker was dropped to 10°C and pre-induction samples were collected. For all growths, a 1 mL time zero-hour (t_0_) sample was collected and dispensed into a 1.5 mL microcentrifuge tube. All samples were centrifuged at 14,800 rpm for 5 min (8°C) using a tabletop temperature-controlled microcentrifuge. The supernatant of all samples was carefully removed with a 1 mL pipette without disturbing the pellet (no lysate was left in the tube). All cell pellets were then stored at −80°C until needed for SDS-PAGE analysis. To induce the cells for expression, the correct amount of IPTG was added corresponding to the concentration being tested. For example, for 0.1 mM IPTG, 50 mL of 100 mM IPTG was added (the stock of IPTG was prepared with ultrapure water). Furthermore, to those growths where betaine was added, 50 mL of 1 M betaine (1 mM final concentration) was added. Once IPTG and betaine added, the growths were set back in the 10°C shaker (220 rpm) and post-induction samples (1 mL) were collected at 24, 44, 48, and 52 h. All collected samples were centrifuged at 14,800 rpm for 5 min (8°C) using a tabletop temperature-controlled microcentrifuge, pellets collected as described above and stored at −80°C until needed for SDS-PAGE analysis.

The expression conditions for the other proteases (AaSPI, AaSPV, and JHA15) differed from AaLT and AaET. Therefore, different time points and a specific set of IPTG concentrations in the presence and absence of betaine were set but utilizing the same growth conditions as above (TB media, 10°C, and 220 rpm shaking). For AaSPI, IPTG concentrations of 0.0125 mM (12.5 mM) and 0.003125 mM (3.125 mM) in the presence and absence of 1 mM betaine were set. The time points collected for AaSPI SDS-PAGE analysis were t_0_ (pre-induction) and t = 48, 96, 144, and 168 h (post-induction). As for AaSPV, IPTG concentrations of 0.025 and 0.00625 mM in the presence and absence of 1 mM betaine were set. The time points collected for AaSPV SDS-PAGE analysis were t_0_ (pre-induction) and t = 48, 120, 148, and 168 h (post-induction). Lastly, for JHA15, 0.025 and 0.00625 mM IPTG in the presence and absence of betaine were set. The time points collected for JHA15 SDS-PAGE analysis were t_0_ (pre-induction) and t = 24, 48, 52, and 72 h (post-induction). A total of 40 replicates for each growth was prepared, corresponding to 10 flasks of the different IPTG concentrations tested in the presence and absence of 1 mM betaine. Pellets were collected as described above and stored at −80°C until needed for SDS-PAGE analysis.

### Growth experiment sample preparation for SDS-PAGE, his-tag staining, and imaging

For SDS-PAGE, samples collected from the growth experiments were processed via sonication. To each pellet, 450 μL of 20 mM TRIS-HCl pH 7.2 buffer was added and a pipette was used to resuspend the pellet. Once the pellet was completely soluble, the bacterial cells were sonicated using the FisherBrand Model 120 Sonic Dismembrator to lyse the cells and release contents into the buffer. The dismembrator was set at an amplitude of 25% and sonication was performed for a total of 10 sec (3x total). To prevent overheating of the sample, which can degrade cell contents, samples were kept on ice in between sonication steps. For example, the t_0_ sample was sonicated for 10 sec, followed by the later postinduction time points, making sure to wipe the tip clean with ultrapure water and a kimwipe in between runs. This ensures that there is no cross contamination between sonication steps.

Preparation of the “total” protein sample included the removal of 20 μL of each sonicated sample and transferred into a labeled microcentrifuge tube treated with 3.4 μL of 6x SDS-dye. The sonicated samples were then centrifuged for 5 min at 14,800 rpm (8°C) using a tabletop temperature-controlled microcentrifuge to separate cell debris and other insoluble content. Carefully, without disturbing the pellet, 20 μL of each centrifuged sample was transferred into a labeled microcentrifuge tube along with 3.4 μL of 6x SDS-dye (these are designated as the “soluble” samples). After collection of both total and soluble samples treated with SDS-dye, they were vortexed and centrifuged on the mini tabletop centrifuge (10 s), followed by heat denaturation in a 90°C heat block for 4 minutes. After boiling, samples were then centrifuged on a mini tabletop centrifuge (10 s) to ensure the loading of all content into 4–12% BIS-TRIS gels (12 well, Invitrogen, cat. #NP0322BOX). To estimate the MW of recombinantly expressed protease bands, 5 μL of the PageRuler PreStained Protein Ladder (Thermo Scientific, cat. #PI26616) was used. All gels were run at 175 volts for 45 min with 1x MES (Invitrogen, cat. #NP0002–02) and stained with the InVision His-Tag In-Gel Stain (Invitrogen, cat. #LC6030), following the manufacturer’s protocol, with some modifications. Briefly, gels were fixed with 100 mL fixing solution (40% ethanol + 10% glacial acetic acid) and microwaved for approximately 1 minute or until boiling. This was followed by 10 min incubation and gentle agitation using a microplate shaker (250 rpm). The fixing solution was then decanted, and 100 mL of ultra-pure water was added and microwaved for approximately 1 minute or until boiling. Gels were then placed back on the microplate shaker (250 rpm) for 5 min. This step was repeated twice to ensure removal of the fixing solution. To stain, all water was removed from the trays and 30 mL of InVision His-tag In-gel Stain was added (enough added to fully cover the gels), followed by microwaving for 45 sec or until boiling. All gels were incubated with the stain on a microplate shaker (350 rpm) for 40 min. To remove non-specific binding of the stain to non his_6_-tagged proteins, a 5 min wash with 100 mL of 20 mM Phosphate buffer pH 7.8 was done. To image the his-stained gels, the BioRad ChemiDoc MP Imaging System (Hercules, CA) with the Ethidium Bromide setting and auto exposure was used. The .raw files from the ChemiDoc were saved and used for analysis.

### ImageJ gel quantification and statistical analysis

To quantify the production of recombinant midgut proteases and determine if any statistical differences exist between total and soluble expression, as well as to determine differences in soluble expression of the various IPTG concentrations tested in the presence and absence of betaine, the ImageJ program was used. ImageJ analysis was slightly modified from procedures available, see [33, 34]. To start, all .raw files obtained from photo documentation of the his-tag-stained gels (see previous section above) were opened in Adobe Photoshop to rotate the gels to align the expression bands horizontally and invert the gels from dark background with white bands (**Fig. S1A**) to white background and dark black bands (**Fig. S1B**). This step is important because ImageJ works more efficiently for dark bands with a white background [33, 35]. Once gels are inverted, they were opened in the ImageJ program, and under the Image tab “Type” was changed to 32-bit to remove RGB coloring. Using the Rectangle tool in ImageJ, a rectangle was drawn around the largest expressed total band and used to select all samples. The box was moved to the t_0_ total timepoint and ensuring that the box aligned with all the bands being analyzed, the band was selected using the “Select First Lane” tool (under the Analyze à Gels à Select First Lane tabs). This was followed by moving the box to the next timepoint and selecting “Select Next Lane” (under the Analyze à Gels à Select Next Lane tabs). After each selection, a number will appear indicating successful selection of the gel bands (**Fig. S1C**). Important note, ImageJ requires that the same size box be used for all gel band selections so that pixel detection and area calculations are estimated properly [35]. To determine pixel areas, the “Plot Lanes” tool (under the Analyze à Gels à Plot Lanes tabs) was selected and a new window with plotted peaks are observed (**Fig. S1D**). From this, the “Line Drawing” tool was selected and used to draw a line from the baseline of each peak. Once drawn, the “Wand (Tracing)” tool was used to click within the defined peak leading to the intensity (or pixel area) for each. The values obtained from ImageJ were then used to create a column plot in Excel of post-induction time (x-axis) vs band intensity (y-axis) (**Tables S1-S5**). Given that at t_0_ (pre-induction) there is no expression of recombinant proteases, the pixel area at this timepoint was used to remove background noise from all other bands. For example, t_0_ (pre-induction) total band intensity was used to remove background from all total post-induction samples, and t_0_ (pre-induction) soluble band intensity was used to remove background from all soluble post-induction samples. The values obtained from Excel were transferred to GraphPad Prism version 10.6.0 for statistical analysis. Data analyzed by unpaired Student’s t-test with mean values ± SEM. Asterisks indicate significant differences (*p < 0.0332; **p < 0.0021; ***p < 0.0002; ****p < 0.0001). To determine significant differences between groups, a one-way ANOVA with repeated measures and Tukey multiple comparisons was employed.

## Results

### Lower temperature alone promotes higher production of soluble recombinant AaLT and AaET

**Figure 1:**
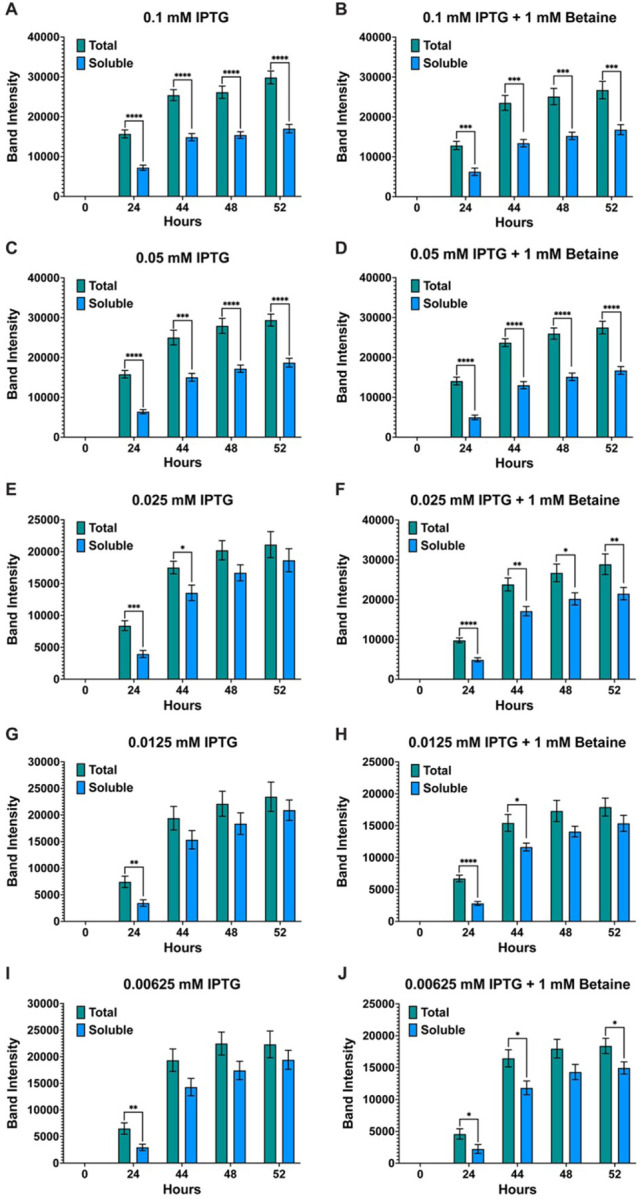
Band intensity comparison between overall (total) and soluble AaLT-Z expression. To determine the effects of lowering the concentration of IPTG on the soluble production of AaLT, column plots were used to compare the band intensity differences between the total and soluble samples of 0.1 mM IPTG (**A & B**), 0.05 mM IPTG (**C & D**), 0.025 mM IPTG (**E & F**), 0.0125 mM IPTG (**G & H**), and 0.00625 mM IPTG (**I & J**) in the presence and absence of 1 mM betaine. As the concentration of IPTG decreases, the more soluble AaLT-Z is observed, reducing the difference between total and soluble samples. Each growth experiment was compiled from 10 individual replicates. Data are presented as mean ± SEM. Statistical significance is represented by stars above each comparison using the unpaired Student’s t test (*p < 0.0332; **p < 0.0021; ***p < 0.0002; ****p < 0.0001) comparing “total” vs “soluble” at each timepoint.

**Figure 2:**
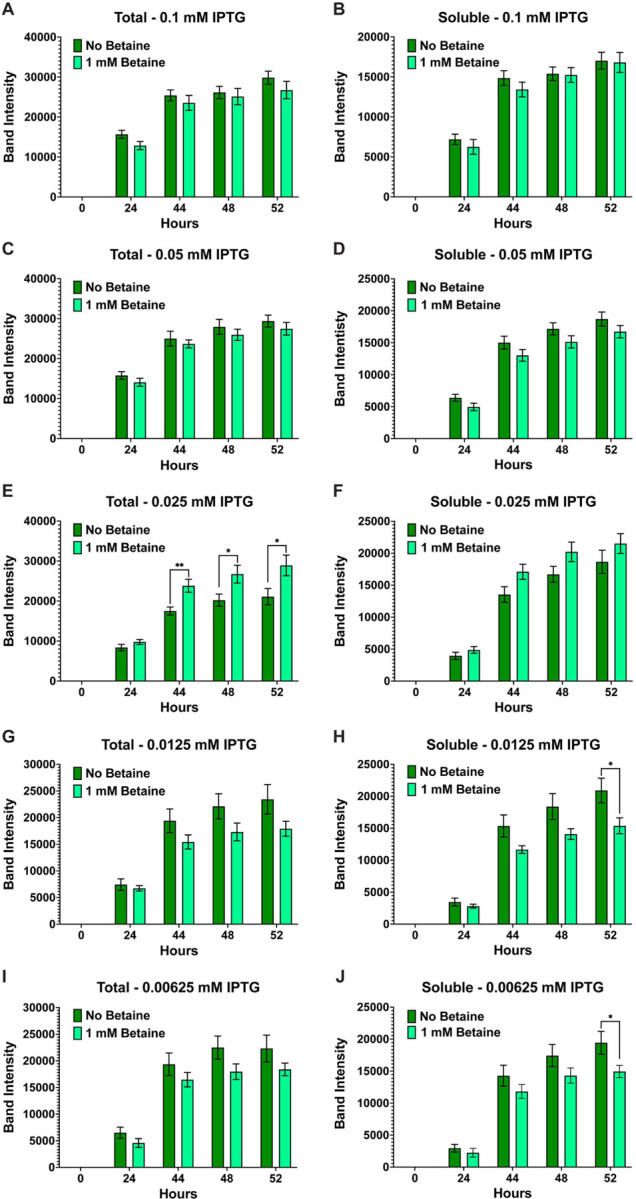
Effect of betaine on the soluble expression of AaLT-Z. The small molecule osmolyte (betaine) is thought to help with preventing aggregation of recombinantly expressed proteins, so (1 mM betaine) was added to the growth experiments of AaLT-Z at the different concentrations of IPTG: 0.1 mM (**A & B**), 0.05 mM (**C & D**), 0.025 mM (**E & F**), 0.0125 mM (**G & H**), and 0.00625 mM (**I & J**). This was done to compare overall (total) AaLT-Z expression in the presence and absence of 1 mM betaine together, as well as soluble AaLT-Z in the presence and absence of 1 mM betaine. Overall, soluble expression is slightly better at 0.0125 and 0.00625 mM IPTG without betaine at the 52 h post-induction timepoint. Each growth experiment was compiled from 10 individual replicates. Data are presented as mean ± SEM. Statistical significance is represented by stars above each comparison using the unpaired Student’s t test (*p < 0.0332; **p < 0.0021) comparing the “no betaine” vs “1 mM betaine” at each timepoint.

Growth experiments for the expression of AaLT using T7 Shuffle cells were set at 10°C and induced with different concentrations of IPTG (0.1, 0.5, 0.025, 0.0125, and 0.00625 mM) in the absence and presence of 1 mM betaine. Visual analysis via SDS-PAGE and his-tag staining led to no visual difference between the growths (**Fig. S2**). Therefore, ImageJ was used to quantify the protein gel bands of all replicate samples, and the band intensity (pixel area obtained) was plotted using GraphPad prism. Overall, the expression of AaLT is much higher at higher concentrations of IPTG, with noticeable differences between total and soluble samples (Fig. 1). For example, at 0.1 and 0.05 mM IPTG, less soluble AaLT is present (Fig. 1A–1D), indicating that rapid transcription/translation is possibly preventing proper folding of the protease. As the IPTG concentration decreases more soluble AaLT is produced and the difference between total and soluble also decreases (Fig. 1E–1J). Interestingly, the small molecule osmolyte betaine did not affect the overall (total) expression and soluble production of AaLT at 0.1–0.025 mM IPTG (Fig. 2A–2F) but did slightly prevent proper folding of AaLT at 0.0125 and 0.00625 mM IPTG (*p < 0.0332, based on unpaired student t test analysis) (Fig. 2G–2J). In fact, overall soluble expression is slightly better at 52 h post-induction for both 0.0125 and 0.00625 mM IPTG without betaine (Fig. 2H and 2J).

**Figure 3:**
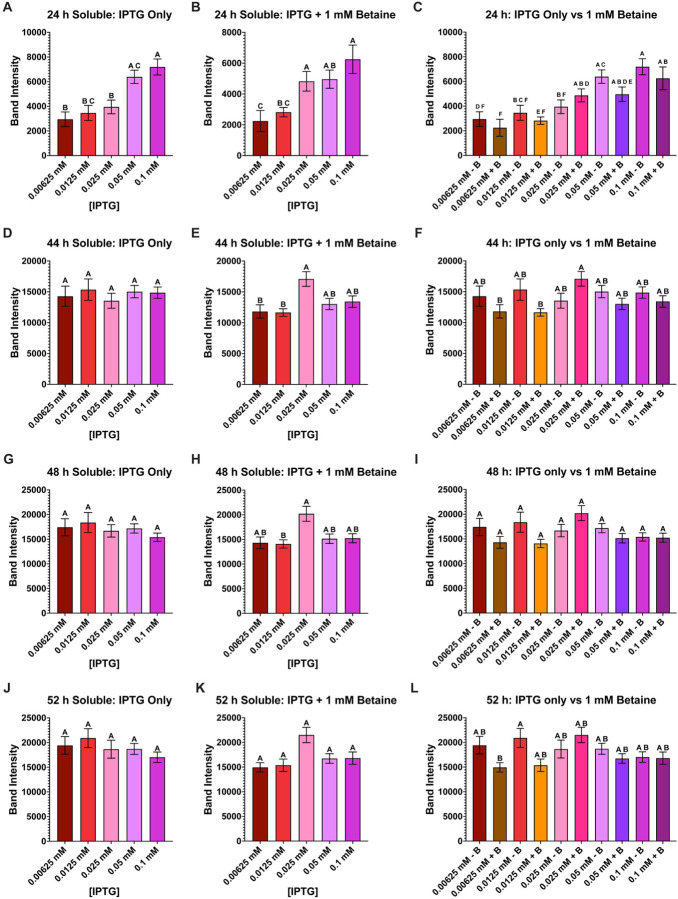
Group comparison of all AaLT-Z soluble samples at each timepoint. Column plots of all soluble samples were used to determine the best conditions that produced the most soluble AaLT-Z: 24 h IPTG only (**A**), 24 h IPTG + betaine (**B**), 24 h IPTG only vs IPTG + betaine (**C**), 44 h IPTG only (**D**), 44 h IPTG + betaine (**E**), 44 h IPTG only vs IPTG + betaine (**F**), 48 h IPTG only (**G**), 48 h IPTG + betaine (**H**), 48 h IPTG only vs IPTG + betaine (**I**), 52 h IPTG only (**J**), 52 h IPTG + betaine (**K**), and 52 h IPTG only vs IPTG + betaine (**L**). The higher concentration of IPTG produces more soluble AaLT-Z at the earlier timepoints but stabilizes at the later timepoints. At 52 h post-induction the highest soluble expression observed is with an IPTG concentration of 0.0125 mM without betaine, but statistically speaking, no overall statistical difference compared to all the other samples except 0.00625 mM IPTG + betaine (which produced the least amount of soluble AaLT-Z). Each growth experiment was compiled from 10 individual replicates. Data are presented as mean ± SEM. Anova analysis with repeated measures and Tukey multiple comparisons was used. Statistically significant differences between the groups are represented by the letters above the columns.

To compare the soluble samples from each IPTG concentration at each timepoint, a one-way Anova analysis with repeated measures and Tukey multiple comparisons was performed (Fig. 3). At 24 h post-induction (regardless, if betaine is present or not) the amount of soluble expression of AaLT is significantly lower at 0.00625 and 0.0125 mM IPTG concentrations, while at 0.025 and 0.05 mM, AaLT soluble expression is statistically lower than 0.1 mM (Fig. 3A–3C). For 44 h post-induction, the most statistically significant amount of soluble AaLT was observed with 0.025 mM IPTG with betaine (Fig. 3E), and the lowest amount was observed with 0.00625 mM IPTG with betaine (Fig. 3F). No significant differences were observed at 48 h post-induction (Fig. 3G–3I). Similarly, when comparing IPTG only samples alone (Fig. 3J) and IPTG + betaine samples alone (Fig. 3K), no statistically significant changes are observed. Only when comparing the band intensities from both groups together is there a statistically noticeable amount of soluble AaLT observed at 52 h post-induction (Fig. 3L). At this timepoint, the highest soluble expression observed is with an IPTG concentration of 0.0125 mM without betaine, but statistically speaking, there is no overall statistical difference compared to all the other samples, except 0.00625 mM IPTG + betaine which has the lowest soluble AaLT production.

**Figure 4:**
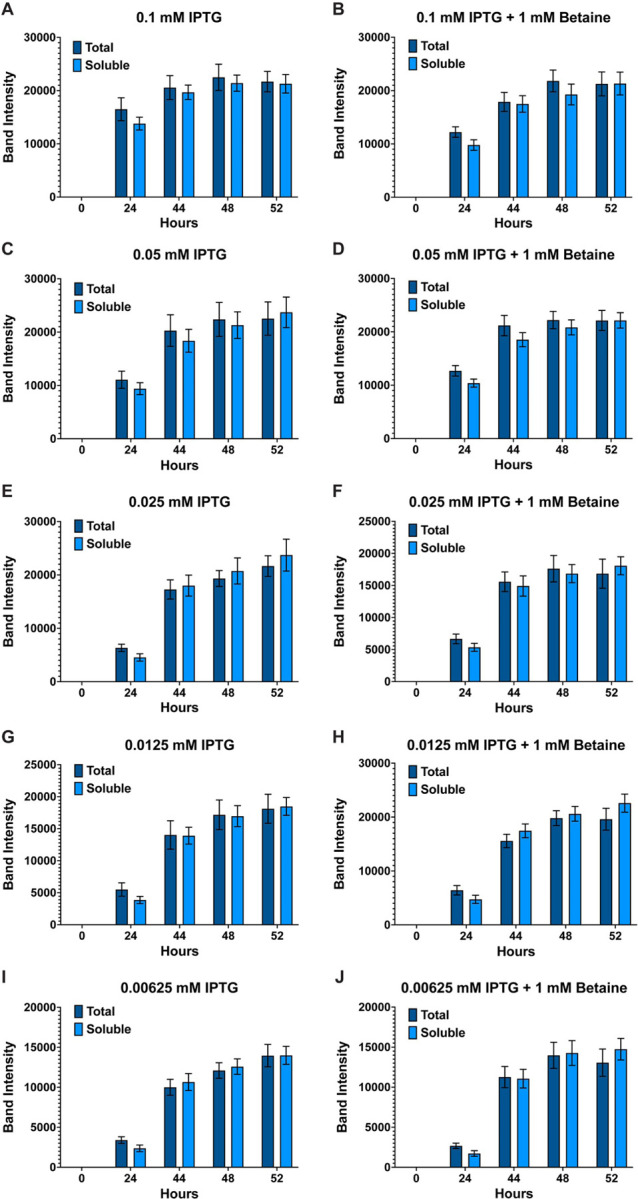
Band intensity comparison between overall (total) and soluble AaET-EK expression. Like the AaLT growths (Fig. 1), the effects of lowering the concentration of IPTG on the soluble production of AaET was determined using column plots. Band intensity differences between total and soluble samples of 0.1 mM IPTG (**A & B**), 0.05 mM IPTG (**C & D**), 0.025 mM IPTG (**E & F**), 0.0125 mM IPTG (**G & H**), and 0.00625 mM IPTG (**I & J**) in the presence and absence of 1 mM betaine were determined. In this case, no statistical differences were observed between total and soluble samples at all IPTG concentrations tested. Each growth experiment was compiled from 10 individual replicates. Data are presented as mean ± SEM. Unpaired Student’s t test was used for analysis.

**Figure 5:**
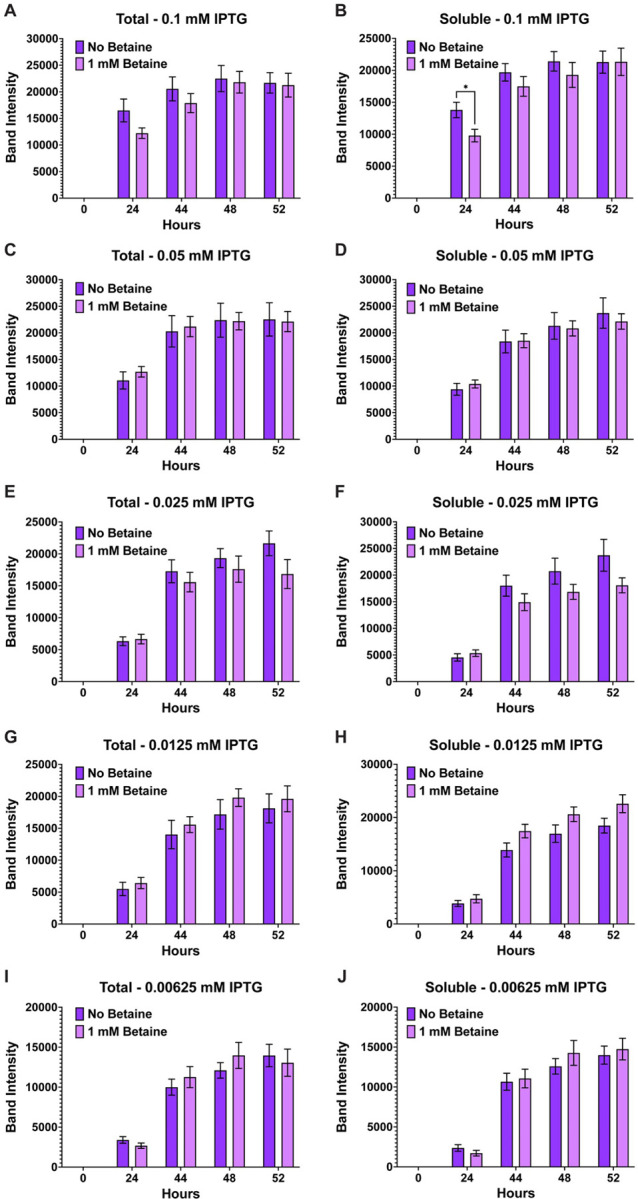
Effect of betaine on the soluble expression of AaET-EK. The effects of betaine on the overall (total) and soluble expression of AaET were compared at all IPTG concentrations: 0.1 mM (**A & B**), 0.05 mM (**C & D**), 0.025 mM (**E & F**), 0.0125 mM (**G & H**), and 0.00625 mM (**I & J**). The osmolyte had no effect on both total and soluble production of AaET. However, at lower IPTG concentrations (0.0125 mM and 0.00625 mM), more soluble expression of AaET-EK is observed compared to the higher IPTG concentrations. Each growth experiment was compiled from 10 individual replicates. Data are presented as mean ± SEM. Statistical significance was determined using the unpaired Student’s t test (*p < 0.0332) comparing “no betaine” vs “1 mM betaine” at each timepoint.

**Figure 6:**
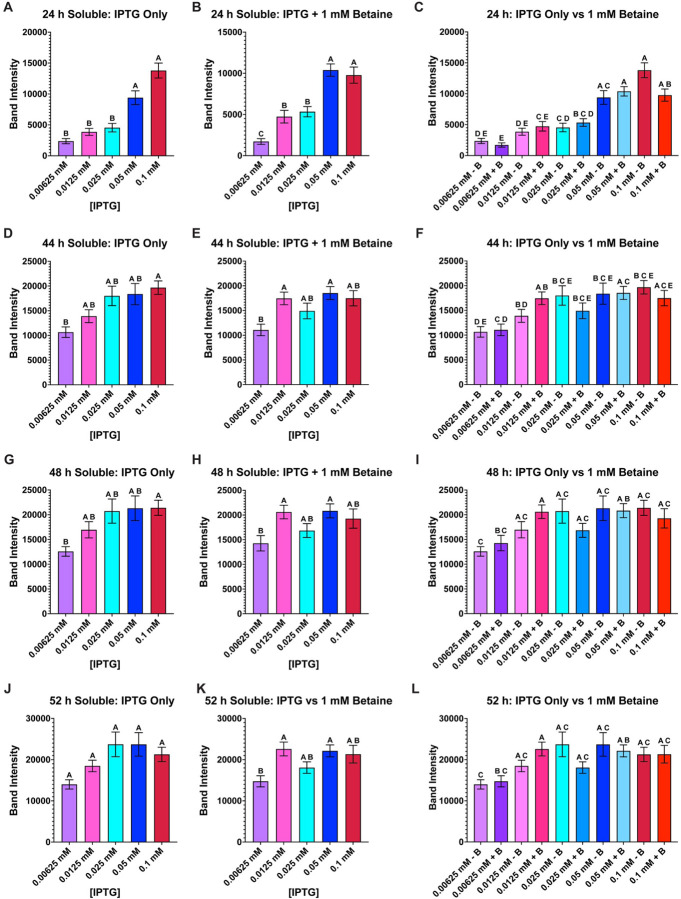
Group comparison of all AaET-EK soluble samples at each timepoint. Column plots of all soluble samples were used to determine the condition that produced the most soluble AaET-EK: 24 h IPTG only (**A**), 24 h IPTG + betaine (**B**), 24 h IPTG only vs IPTG + betaine (**C**), 44 h IPTG only (**D**), 44 h IPTG + betaine (**E**), 44 h IPTG only vs IPTG + betaine (**F**), 48 h IPTG only (**G**), 48 h IPTG + betaine (**H**), 48 h IPTG only vs IPTG + betaine (**I**), 52 h IPTG only (**J**), 52 h IPTG + betaine (**K**), and 52 h IPTG only vs IPTG + betaine (**L**). The higher concentration of IPTG produces more soluble AaET-EK at the earlier timepoints but stabilizes at the later timepoints. At52 h post-induction the highest soluble expression observed is with IPTG concentrations of at 0.0125 mM IPTG + betaine, 0.025 mM IPTG without betaine, and 0.05 mM IPTG without betaine. Each growth experiment was compiled from 10 individual replicates. Data are presented as mean ± SEM. Anova analysis with repeated measures and Tukey multiple comparisons was used. Statistically significant differences between the groups are represented by the letters above the columns.

To test if lowering the IPTG concentration in the presence and absence of betaine would help improve the soluble expression of AaET, growth experiments with the same conditions used for AaLT were performed. Samples analyzed via SDS-PAGE and his-staining had no visual differences between the AaET growths at the different IPTG concentrations (+/−betaine) (**Fig. S3**). In addition, quantifying the protein gel bands led to no significant differences between total and soluble samples at 0.1 mM and all the IPTG concentrations tested, regardless if betaine was added (Fig. 4 & 5). This indicates that the lower 10°C temperature alone allowed AaET to properly fold, unlike for AaLT where significant differences between total and soluble samples were observed (compare with Fig. 1 & 2). However, lower overall soluble production of AaET is observed at 0.0125 mM (Fig. 5H) and 0.00625 mM IPTG (Fig. 5J) compared to the higher IPTG concentrations (Fig. 5B, 5D, 5F). To confirm this, a one-way Anova with repeated measures and Tukey multiple comparisons was used to compare the soluble samples from each IPTG concentration at each timepoint with each other. The results do confirm that 0.00625 mM (+/−betaine) and 0.0125 mM IPTG without betaine have the lowest overall soluble production of AaET, but the growths with 0.0125 mM IPTG + betaine statistically produced more AaET than both (Fig. 6). More importantly, at the latest timepoint (52 h post-induction), where the highest bacterial density is achieved, overall AaET production seems to be the highest at 0.0125 mM IPTG + betaine, 0.025 mM IPTG without betaine, and 0.05 mM IPTG without betaine, but statistically speaking, these are not significantly different when comparing all growths (Fig. 6L). The group comparison confirms that the lowest production of AaET is with growths induced with 0.00625 mM (+/− betaine) and 0.0125 mM IPTG without betaine.

### Soluble recombinant expression of AaSPV, AaSPI and JHA15 benefit from lower than 0.05 mM IPTG concentrations

**Figure 7:**
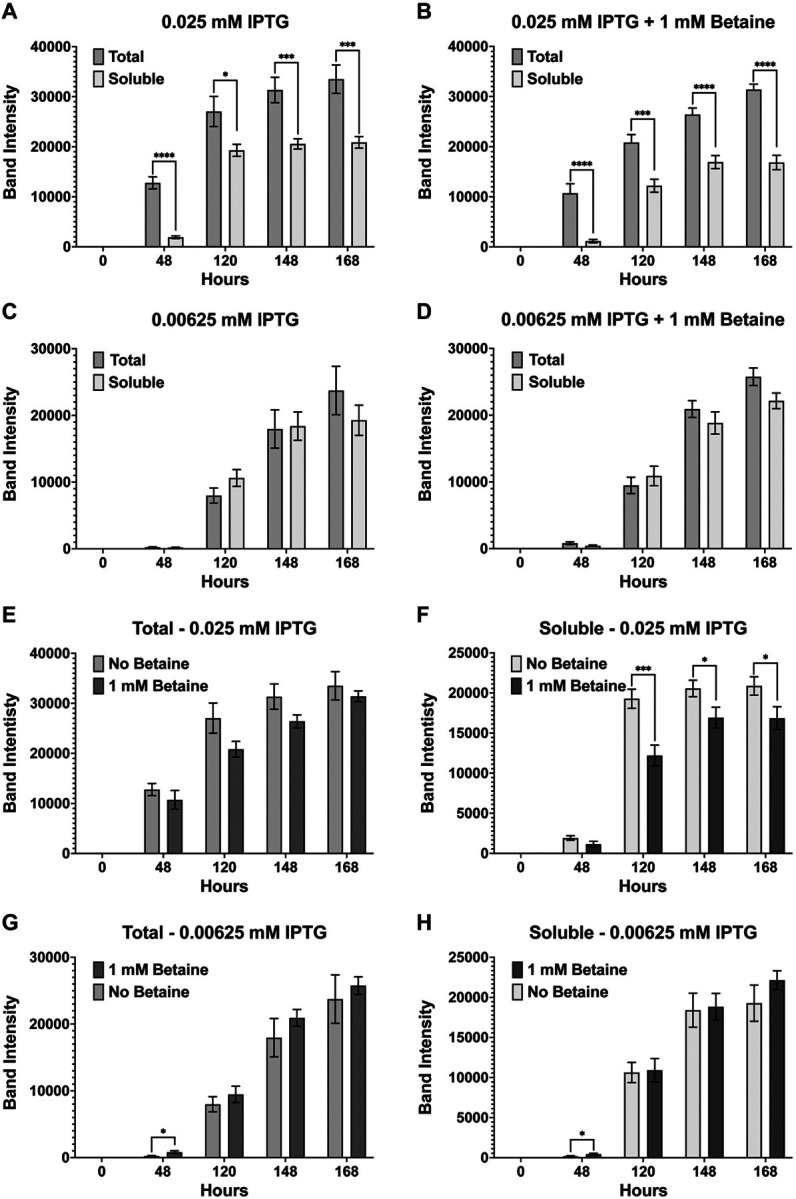
Band intensity comparison between overall (total) versus soluble AaSPV-EK expression and effect of betaine. Extending the growth period beyond 52 h and lowering the IPTG concentration to 0.025 mM (25 mM) led to soluble production of AaSPV (**A & B**), but not as much as 0.00625 mM (6.25 mM) IPTG (**C & D**). Statistically significant differences between “total” and “soluble” samples for each timepoint at 0.025 mM IPTG with or without betaine are observed, indicating that not all produced AaSPV is soluble. In comparing the effect of betaine, total samples at 25 mM IPTG (**E**) and 6.25 mM IPTG (**G**) had no effect on overall expression of AaSPV. However, betaine affected folding of AaSPV at 25 mM IPTG (**F**) but not at 6.25 mM IPTG (**H**). Data are presented as mean ± SEM. Statistical significance is represented by stars above each comparison using the unpaired Student’s t test (*p < 0.0332; **p < 0.0021; ***p < 0.0002; ****p < 0.0001) comparing “total” vs “soluble” at each timepoint, as well as “no betaine” vs “1 mM betaine”.

**Figure 8:**
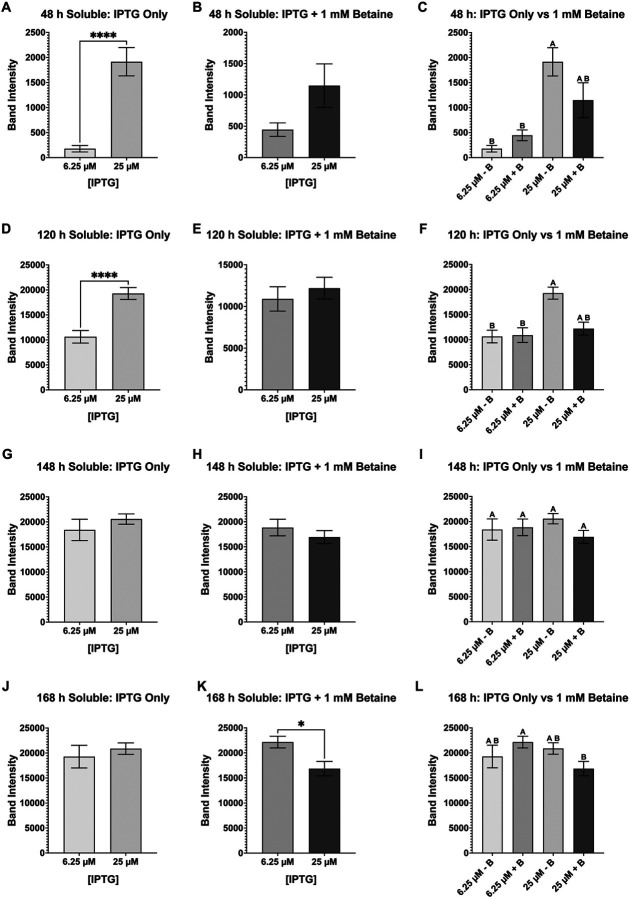
Pair and group comparison of AaSPV-EK soluble samples at each timepoint. Column plots of all soluble samples were used to determine the best conditions that produced the most soluble AaSPV-EK: 48 h IPTG only (**A**), 48 h IPTG + betaine (**B**), 48 h IPTG only vs IPTG + betaine (**C**), 120 h IPTG only (**D**), 120 h IPTG + betaine (**E**), 120 h IPTG only vs IPTG + betaine (**F**), 148 h IPTG only (**G**), 148 h IPTG + betaine (**H**), 148 h IPTG only vs IPTG + betaine (**I**), 168 h IPTG only (**J**), 168 h IPTG + betaine (**K**), and 168 h IPTG only vs IPTG + betaine (**L**). The higher concentration of IPTG produces more soluble AaSPV-EK at the earlier timepoints (48 and 120 h) but stabilizes at the later timepoints (148 and 168 h). At 168 h post-induction the highest soluble expression observed is with an IPTG concentration of 6.25 mM with betaine, but statistically speaking, no overall statistical difference compared to all the other samples, except 25 mM IPTG + betaine (which produced the least amount of soluble AaSPV-EK). Each growth experiment was compiled from 10 individual replicates. Data are presented as mean ± SEM. For pairwise comparisons, the unpaired Student’s t test was used (*p < 0.0332; ****p < 0.0001). For the grouped analysis, Anova with repeated measures and Tukey multiple comparisons was used. Statistically significant differences between the groups are represented by the letters above the columns.

Growth experiments were repeated for two never recombinantly expressed midgut proteases (AaSPV and AaSPI) (Brackney et al 2010), and JHA15 which was first cloned and expressed using BL21(DE3)pLysS cells (Bian et al 2008). Initial growth experiments with 0.1 and 0.05 mM IPTG led to mostly insoluble production of all three midgut proteases (**Fig. S4**). Therefore, growths were repeated with 0.025 mM (25 mM) and 0.00625 mM (6.25 mM) IPTG in the presence and absence of betaine for AaSPV and JHA15. Unfortunately, these concentrations were still too high to allow for the soluble production of AaSPI, so the concentration of IPTG was lowered to 3.25 mM and 12.5 mM IPTG (+/−betaine). Furthermore, growths were extended longer than 52 h to ensure a higher density of bacterial cells to determine if the lower IPTG concentrations under these conditions would express soluble proteases. Given these conditions, for AaSPV, overall expression was slightly higher at 0.025 mM IPTG than 0.00625 mM, regardless if betaine was added (Fig. 7). At 0.025 mM IPTG, less soluble production of AaSPV is observed at all timepoints tested, indicating that this concentration was still relatively high leading to rapid transcription/translation preventing proper folding of the protease (Fig. 7A & 7B). For 0.00625 mM IPTG, no statistical differences between total and soluble were observed, which indicates that most of the production of AaSPV is properly folded (Fig. 7C & 7D). Interestingly, the small molecule osmolyte betaine did not affect the overall expression of AaSPV (Fig. 7E & 7G) but did affect folding and soluble protease production in the presence of betaine. For example, lower soluble AaSPV was observed at 0.025 mM IPTG with betaine (Fig. 7F). However, at 0.00625 mM IPTG no statistical differences were observed between the no betaine and + betaine growths (Fig. 7H). Next, we compared the soluble samples from each IPTG concentration at each timepoint using the unpaired student t-test (Fig. 8). At 48 and 120 h post-induction the amount of soluble expression of AaSPV is significantly lower at the 0.00625 mM (6.25 mM) IPTG only samples compared to 0.025 mM (25 mM) IPTG only samples (Fig. 8A & 8D) with no differences observed with the IPTG + betaine samples (Fig. 8B & 8E). The only other slightly significant difference was between 0.00625 mM (6.25 mM) IPTG compared to 0.025 mM (25 mM) + betaine at 168 h post-induction (Fig. 8K). Lastly, the group of each concentration (+/− betaine) at each timepoint was compared using a one-way Anova analysis with repeated measures and Tukey multiple comparisons. Not surprising, at 24 h post-induction, the lower the IPTG concentration the lower the soluble production of AaSPV, but as time progresses, the amount of soluble protease increases (Fig. 8C, 8F, 8I, & 8L). At 144 h post-induction no statistical differences are observed in the amount of soluble AaSPV produced. However, at 168 h the lowest observable production of AaSPV was with 0.025 mM (25 mM) IPTG + betaine.

**Figure 9:**
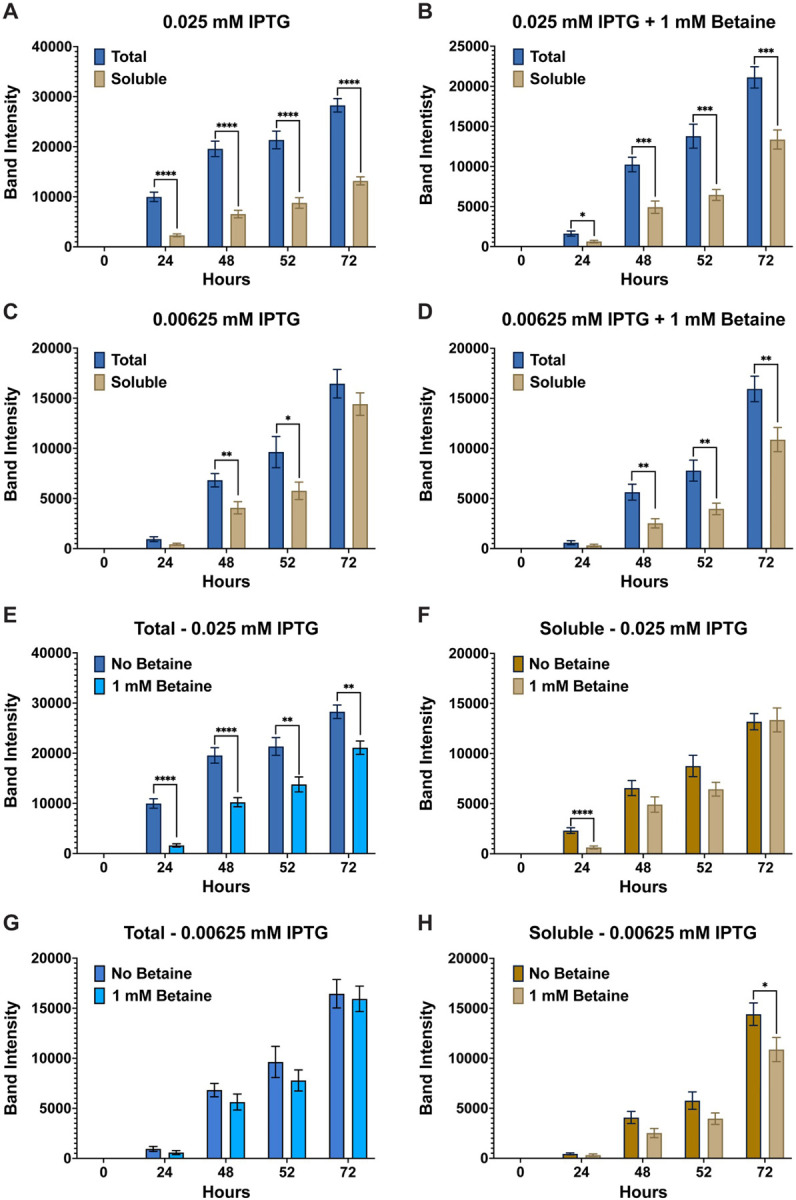
Band intensity comparison between overall (total) versus soluble JHA15-EK expression and effect of betaine. Of all proteases to solubly express, JHA15-EK was the most difficult. Lowering the IPTG concentration to 0.025 mM (25 mM) (**A & B**) and 0.00625 mM (6.25 mM) IPTG (**C & D**), while extending the growth period to 72 h, still did not lower the statistical difference between total and soluble production of the protease. Statistically significant differences between each condition were still observed, indicating that not all produced JHA15-EK is soluble. Further, unlike other proteases in this study, betaine affected overall (total) expression when cells induced with 25 mM IPTG (**E**), and only differences at 24 h post-induction were observed for the soluble samples at 25 mM IPTG (**F**). Betaine had no effect on overall (total) expression at 6.25 mM (**G**) but did lead to a statistical difference at 72 h post-induction, with betaine affecting folding of the protease (**H**). Data are presented as mean ± SEM. Statistical significance is represented by stars above each comparison using the unpaired Student’s t test (*p < 0.0332; **p < 0.0021; ***p < 0.0002; ****p < 0.0001) comparing “total” vs “soluble” at each timepoint, as well as “no betaine” vs “1 mM betaine”.

**Figure 10:**
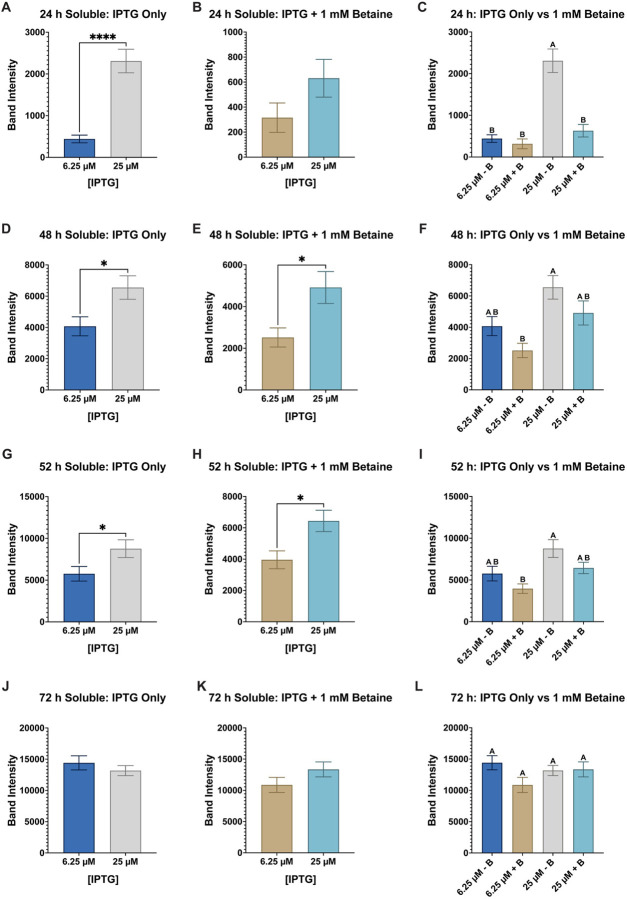
Pair and group comparison of JHA15-EK soluble samples at each timepoint. Column plots of all soluble samples were used to determine the best conditions that produced the most soluble JHA15-EK: 24 h IPTG only (**A**), 24 h IPTG + betaine (**B**), 24 h IPTG only vs IPTG + betaine (**C**), 48 h IPTG only (**D**), 48 h IPTG + betaine (**E**), 48 h IPTG only vs IPTG + betaine (**F**), 52 h IPTG only (**G**), 52 h IPTG + betaine (**H**), 52 h IPTG only vs IPTG + betaine (**I**), 72 h IPTG only (**J**), 72 h IPTG + betaine (**K**), and 72 h IPTG only vs IPTG + betaine (**L**). The higher concentration of IPTG produces more soluble JHA15-EK at all timepoints except at 72 h post-induction. At this timepoint, no overall statistical difference in soluble expression is observed. Each growth experiment was compiled from 10 individual replicates. Data are presented as mean ± SEM. For pairwise comparisons, the unpaired Student’s t test was used (*p < 0.0332; ****p < 0.0001). For the grouped analysis, Anova with repeated measures and Tukey multiple comparisons was used. Statistically significant differences between the groups are represented by the letters above the columns.

As with the other growths, the overall expression of JHA15 was slightly higher with the higher IPTG concentration, in this case 0.025 mM (25 mM) IPTG (Fig. 9). However, at both concentrations tested there were still significant differences between total and soluble (Fig. 9A–9D), with only the 72 h post-induction timepoint at 0.00625 mM (6.25 mM) IPTG only having no statistical difference between the two (Fig. 9C). This indicates that the overall production of JHA15 is soluble and properly folded at this timepoint. Unlike the AaSPV growth, betaine negatively affected overall JHA15 production at 0.025 mM (25 mM) IPTG (Fig. 9E) but had no statistical difference on soluble production (Fig. 9F). Alternatively, at 0.00625 mM (6.25 mM) IPTG, overall JHA15 production was not affected by betaine (Fig. 9G) and only a slight difference was observed at the 72 h timepoint. At this timepoint, betaine slightly affected the folding of JHA15 (Fig. 9H). In comparing all soluble samples, statistical differences were observed at most of the earlier timepoints up to 52 h post-induction but stabilized at the final timepoint collected at 72 h (Fig. 10). This indicates that soluble production is not statistically different regardless of the IPTG concentration or presence of betaine at this last timepoint collected. Differing from the results observed for AaLT, AaET, and AaSPV.

**Figure 11:**
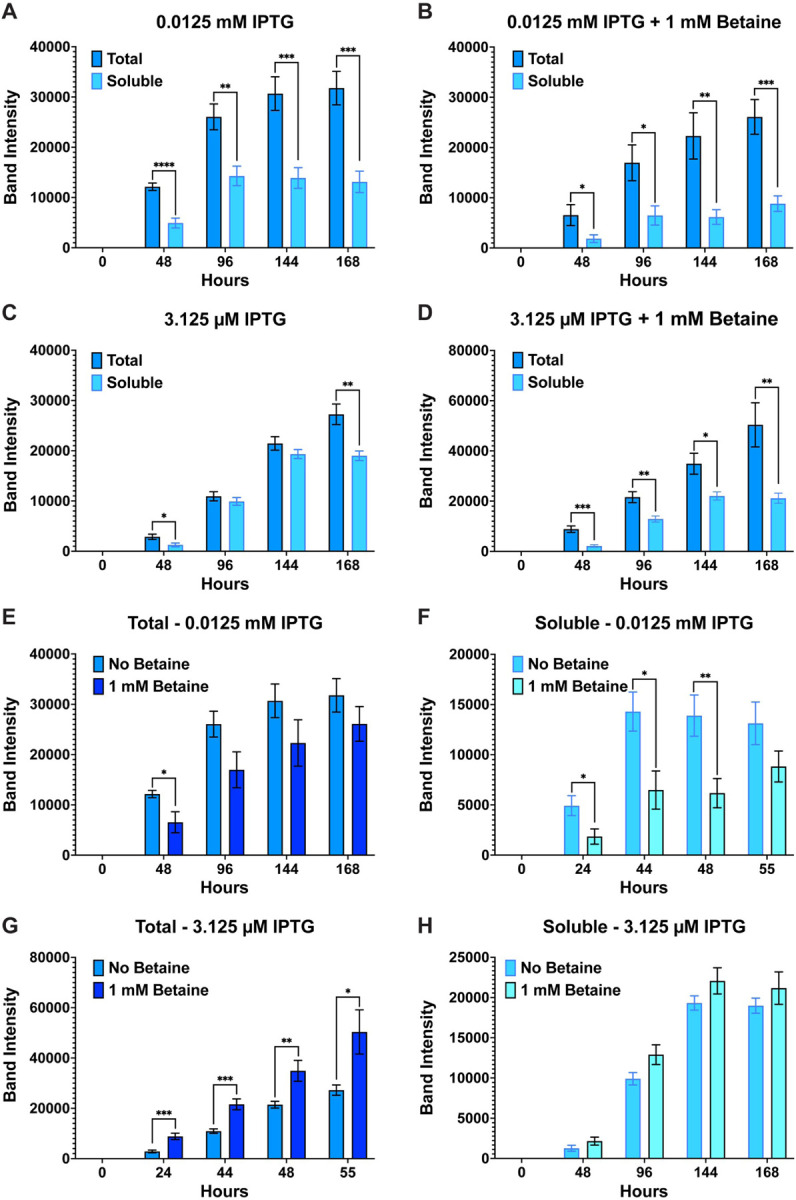
Band intensity comparison between overall (total) versus soluble AaSPI-NL expression and effect of betaine. AaSPI-NL was just as difficult to solubly express as JHA15, but lowering the concentrations below 0.025 mM (25 mM) proved to help produce soluble protease. At 0.0125 mM (12.5 mM) IPTG (**A & B**) and 3.125 mM IPTG (**C & D**), soluble AaSPI-NL was observed. However, statistically significant differences are observed between the total and soluble samples at each IPTG concentration, indicating that not all produced AaSPI-NL is soluble. Further, betaine did not affect overall protease production at 12.5 mM IPTG at the later timepoints (**E**) but did affect soluble production when compared to the “no betaine” samples (**F**). At the lower (3.125 mM) IPTG concentration, betaine affected the overall (total) production of AaSPI (**G**) but no statistical difference was observed on the soluble production of the protease (**H**). Data are presented as mean ± SEM. Statistical significance is represented by stars above each comparison using the unpaired Student’s t test (*p < 0.0332; **p < 0.0021; ***p < 0.0002; ****p < 0.0001) comparing “total” vs “soluble” at each timepoint, as well as “no betaine” vs “1 mM betaine”.

**Figure 12:**
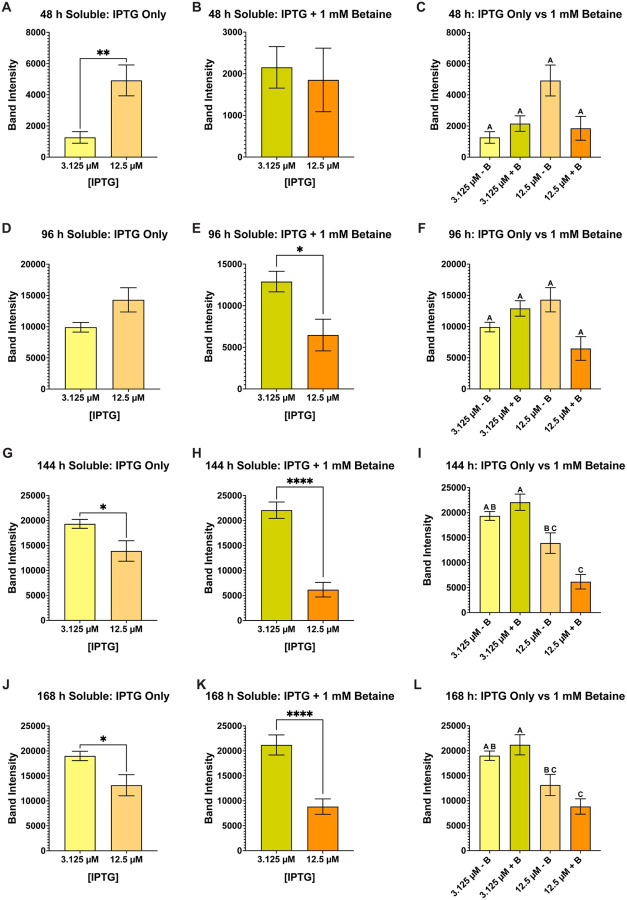
Pair and group comparison of AaSPI-NL soluble samples at each timepoint. Column plots of all soluble samples were used to determine the best conditions that produced the most soluble AaSPI-NL: 48 h IPTG only (**A**), 48 h IPTG + betaine (**B**), 48 h IPTG only vs IPTG + betaine (**C**), 96 h IPTG only (**D**), 96 h IPTG + betaine (**E**), 96 h IPTG only vs IPTG + betaine (**F**), 144 h IPTG only (**G**), 144 h IPTG + betaine (**H**), 144 h IPTG only vs IPTG + betaine (**I**), 168 h IPTG only (**J**), 168 h IPTG + betaine (**K**), and 168 h IPTG only vs IPTG + betaine (**L**). Overall, the lower IPTG concentration (3.125 mM IPTG) produced statistically more soluble AaSPI-NL than 12.5 mM IPTG after 96 h post-induction. At 144 and 168 h post-induction, 3.125 mM with betaine produced the most soluble AaSPI-NL, but not so statistically different than 3.125 mM without betaine. Each growth experiment was compiled from 10 individual replicates. Data are presented as mean ± SEM. For pairwise comparisons, the unpaired Student’s t test was used (*p < 0.0332; **p < 0.0021; ****p < 0.0001). For the grouped analysis, Anova with repeated measures and Tukey multiple comparisons was used. Statistically significant differences between the groups are represented by the letters above the columns.

Of all the mosquito midgut proteases recombinantly expressed in this study, AaSPI has been the most difficult to solubly produce. Concentrations of IPTG as low as 3.125 mM and 12.5 mM had to be utilized to be able to detect soluble AaSPI (**Supplementary Table 5 file**). Even so, at both these IPTG concentrations (+/− betaine) notable significant differences were observed between the total and soluble samples indicating that not all expressed AaSPI was properly folded (Fig. 11A - 11D). At 12.5 mM IPTG, betaine negatively affected the overall expression of AaSPI (Fig. 11E), as well as the overall soluble production of the protease (Fig. 11F). Alternatively, at the lower IPTG concentration of 3.125 mM, betaine increased overall AaSPI production (Fig. 11G) but had no overall statistical effect on folding of AaSPI, both producing nearly the same amount of soluble protease (Fig. 11H). In Fig. 12, we compared all the soluble samples with each other, beginning with an unpaired student t-test to compare each IPTG concentration with or without betaine. As the results indicate, only the 48 h samples with IPTG + betaine (Fig. 12B) and the 96 h samples without betaine (Fig. 12D) were the only two conditions that did not have any statistical differences between them, the cells produced nearly the same amount of soluble AaSPI at these timepoints. However, when comparing the groups at all timepoints collected, statistical differences in the amount of soluble AaSPI are observed at 144 h (Fig. 12I) and 168 h (Fig. 12L) postinduction, with 12.5 mM IPTG + betaine having the lowest amount of soluble protease at both timepoints. The growth samples without betaine at 12.5 mM IPTG had the next lowest compared to the samples induced with 3.125 mM IPTG (+/− betaine). The other two timepoints (48 h (Fig. 12C) and 96 h (Fig. 12F)) had not statistical differences between them.

## Discussion

Recombinant protein expression in *E. coli* remains a widely used and effective method for producing high amounts of heterologous proteins. However, when post-translational modifications are needed (*e.g*., disulfide bond formation) expression in wildtype *E. coli* can hinder proper protein folding due to its reducing cytoplasm, the site of transcription/translation [3–6]. This limitation was a major factor in early attempts of recombinantly expressing *Ae. aegypti* midgut proteases using BL21(DE3) and its derivatives [26]. *Ae. aegypti* mosquito proteases typically contain anywhere between 2–4 disulfide bonds [2, 13, 14, 26] and the reducing cytoplasm inhibits proper formation of this post-translational modification. With the advent of T7 SHuffle cells, which have a more oxidizing cytoplasm [29], the production of recombinant mosquito midgut proteases dependent on disulfide bonds was improved [2]. However, the use of these cells alone did not immediately lead to the soluble production of these midgut proteases. Media and growth temperature conditions had to be altered to allow for proper folding. By lowering the temperature and extending the growth period enabled the soluble expression of four midgut proteases (AaSPVI, AaSPVII, AaLT, and AaET). Although successful, AaLT and AaET were not as abundantly produced compared to the other two proteases. Therefore, to improve soluble production of these two proteases in *E. coli*, we focused on further modifying the regulation in the amount of T7 RNA polymerase by reducing the concentration of IPTG.

The small-molecule inducer *isopropyl-*β*-D-thiogalactopyranoside* (IPTG) is not metabolized by bacterial cells, keeping the concentration constant throughout the whole growth expression period, and at higher concentrations can lead to rapid expression of T7 RNA polymerase leading to rapid transcription and simultaneous translation of recombinant proteins [3, 6, 31, 36–38]. This rapid rate of simultaneous transcription and translation may prevent proper folding of heterologous expressed proteins in bacteria.

In fact, this was the case observed with the recombinant expression of AaLT. In this study, at higher concentrations of IPTG (> 0.025 mM) a statistical difference between overall (total) and soluble expression was observed, with more insoluble protease produced with the higher IPTG concentrations. However, as the IPTG concentration was decreased, the difference between overall (total) and soluble AaLT production also decreased, indicating that most of the protein produced had properly folded. The lower IPTG concentration, along with the colder temperature (10°C), reduced the rate of transcription/translation producing overall lower amounts of AaLT. By producing lower amounts of protease, this lowers AaLT protein accumulation, and therefore aggregation. Furthermore, extending the growth period to 52 h post-induction increases the bacterial density to be able to produce more soluble AaLT over time. Similar results were observed with AaSPV, JHA15, and AaSPI. Inducing at higher concentrations of IPTG led to higher statistical differences between overall (total) and soluble expression of the proteases. However, in these cases, IPTG concentrations of 0.025 mM (25 mM) (for AaSPV and JHA15), 0.00625 mM (6.25 mM) (AaSPV and JHA15), and as low as 3.125 mM (for AaSPI) had to be utilized. These results were surprising given that AaSPV and AaSPI also contain only three disulfide bonds, while JHA15 contains only two disulfide bonds. AaLT (and the other successfully solubly expressed proteases, AaSPVI, AaSPVII, and AaET) all contain three disulfide bonds, which should not have affected folding as much during bacterial expression. A more likely explanation is that the accumulation of AaSPV, JHA15, and AaSPI in the bacterial cell may have been toxic, which initiates the inclusion body response and encapsulating the proteases to prevent cell death or growth arrest [3, 6, 36, 37]. Most of the midgut proteases studied to date (AaET, AaSPVI, AaSPVII, and AaCHYMO) have been shown to autoactivate in the cell during recombinant bacterial protein expression [2, 13, 14]. So, it is likely that these proteases may be autoactivating and the activity may be causing damage to the bacteria. Regardless of this effect, lowering the IPTG concentration at or below 0.025 mM (25 mM) does produce soluble AaSPV, JHA15, and AaSPI proteases, helping overcome the possible toxicity of protease accumulation. Once we begin the large-scale expression and purification of these three proteases, will we be able to determine if they also autoactivate like AaET, AaSPVI, AaSPVII, and AaCHYMO.

Surprisingly, no statistical difference was observed between the overall (total) and soluble production of AaET across all IPTG concentrations tested. This indicates that the protease produced was nearly soluble at all conditions. The only obvious difference is in the amount of overall AaET expression. For example, lower IPTG concentrations reduced overall (total) protein expression leading to lower yields of soluble AaET. This is explained by the fact that at low IPTG concentrations, lower transcription of T7 RNA polymerase is initiated, producing less polymerase enzyme, which leads to slower transcription of AaET. The higher the concentration of IPTG, the more inducer is available to bind to the lac repressor in more cells (freeing up the promoter region) and initiate transcription/translation of T7 RNA polymerase leading to more AaET production. This was completely opposite of what was observed with AaLT, AaSPV, JHA15, and AaSPI, where the higher concentrations of IPTG led to more insoluble protease than at low concentrations of IPTG. In the case of AaET, temperature played a more important role than the IPTG concentration. When AaET was first solubly expressed, an IPTG concentration of 0.1 mM and growth temperature conditions at 15° and 23°C were set, with the latter temperature leading to autoactivation of the protease and the former not producing enough soluble AaET [2]. The 10°C colder temperature not only slowed down the rate of transcription/translation but also prevented autoactivation of the protease during bacterial growth expression.

In parallel with these studies, the small molecule osmolyte betaine was added to the growth experiments to assess its effect on the soluble expression of mosquito midgut proteases. With the difficulty in solubly expressing mosquito midgut proteases, we searched the literature for methods that would help improve folding and solubility. As mentioned above, this led to the changing of bacterial cell strains, the use of richer media, and the lowering of bacterial growth temperature. Another approach suggested is the addition of osmolytes, like betaine, which help *E. coli* manage the increase in osmotic pressure caused by the accumulation of recombinant protein production in the cell. Additionally, osmolytes are thought to also act as “molecular chaperones” thereby assisting in protein folding and leading to increased solubility [3, 32, 37]. With the addition of 1 mM betaine to the growth conditions tested (as suggested by the literature reviews), no real soluble expression pattern was observed for all proteases. Betaine affected each protease differently. For AaLT, no differences between overall (total) and soluble expression were observed at 0.05 and 0.1 mM IPTG, but at lower concentrations (^3^ 0.025 mM IPTG) betaine produced slightly less soluble AaLT. Yet for AaET, there were no differences between overall (total) and soluble expression at all conditions tested. With AaSPV, betaine affected the soluble production of the protease at 0.025 mM (25 mM) IPTG, but for JHA15, only overall (total) protein expression was affected at the same IPTG concentration. Interestingly, for AaSPI, betaine affected overall (total) expression of the protease at 3.125 mM, with no statistical difference between the soluble production of the protease at the same concentration. However, at 12.5 mM IPTG, betaine affected the soluble production of AaSPI. Unfortunately, the mechanism by which osmolytes (like betaine) stabilize recombinant proteins is still not fully understood [32], so it is not surprising that the effects of betaine in this study are all different. One consistent observation was that betaine had more pronounced effects at higher IPTG concentrations. This could be because as recombinant protein accumulates in the cell, the osmotic pressure also increases, triggering the cell to utilize the osmolyte to balance the pressure change. In this context, osmolyte accumulation may destabilized unfolded proteins thereby promoting the native folded state [3, 32, 37]. However, more work on the effects of betaine and other small molecule osmolytes are needed to determine the exact stabilization mechanism.

## Conclusions

The goal of this work was to determine if decreasing the inducer concentration (IPTG) affects the soluble production of *Ae. aegypti* mosquito midgut proteases. In previous studies, we found that utilizing the correct bacterial strain, the use of richer culture media, and lowering the induction temperature led to the soluble production of four midgut proteases [2]. However, not all proteases were equally solubly expressed. Therefore, this study focused on testing lower IPTG concentrations at low temperature (10°C) to further slow the rate of transcription and translation of recombinantly expressed mosquito midgut proteases in bacteria. Indeed, lower IPTG concentrations do promote proper folding in all proteases but AaET. With AaET, temperature was the only important factor that led to the soluble production of the protease. Furthermore, we attempted to determine if a small molecule osmolyte (betaine) would influence the soluble production of mosquito midgut proteases. In our study, betaine seemed to be more important at higher concentrations of IPTG than at low concentrations. However, more studies focusing on betaine and other osmolytes are needed to better understand their stabilization mechanism. More importantly, this work has shown the advantages of lowering the inducer concentration at low temperature to produce difficult to solubly express proteases dependent on disulfide bond formation in *E. coli*. This study provides a blueprint for researchers who have never attempted IPTG concentrations lower than 0.1 mM to recombinantly express proteins in bacteria.

## Supplementary Material

Supplementary Files

This is a list of supplementary files associated with this preprint. Click to download.

- SupplementaryTable1AaLTGrowthExperimentsImageJAnalysiswithproteingels.xlsx
- SupplementaryTable2AaETGrowthExperimentsImageJAnalysiswithproteingels.xlsx
- SupplementaryTable3AaSPVGrowthExperimentsImageJAnalysiswithproteingels.xlsx
- SupplementaryTable4JHA15GrowthExperimentsImageJAnalysiswithproteingels.xlsx
- SupplementaryTable5AaSPIGrowthExperimentsImageJAnalysiswithproteingels.xlsx
- SupplementaryFile1RawFilesofGelsforSupplFigures.pdf
- SupplementaryFigure1.tif
- SupplementaryFigure2.tif
- SupplementaryFigure3.tif
- SupplementaryFigure4.tif

## Figures and Tables

**Table 1.** AaSPI, AaSPV, and JHA15 Forward and Reverse primers used for amplification of the genes of interest. The primers were purchased from ELIM Biopharmaceuticals, Inc. (Hayward, CA). The melting temperature (TM) of each primer was determined by the company. The restriction enzymes for each primer are underlined and in bold, while the heterologous enterokinase sequence is italicized and in bold.

Gene Construct	Primer	Primer Sequence	T_m_ (°C)
**AaSPI Zymogen (WT) No Leader**	AaSPI-No Leader-Fwd	5’-AAAAA**CATATG**TACACTTGCAAATCTACCGGTAATAC-3’	64.68
	AaCHYMO - Zym-pET-Rev 1	5’-AAAAA**AAGCTT**ATTATTCTAGGAATGACTGCACCCAT-3’	64.68
			
**AaSPVEK**	AaSPV-mEK-Fwd	5’-AAAAA**CATATG*****AACAACAACCTCGGCGATGACGATGACAAGATC***ATCGGCGGTTTTCCC-3’	75.02
	AaSPV-Rev	5’-AAAAA**CTCGAG**TTATTAAACTGTGAATTTCACATTTTCTC-3’	66.96
			
**JHA15-EK**	JHA15-EK-Fwd	5’-AAAAA**CATATG*****AACAACAACCTCGGCGATGACGATGACAAGATC***GTCGGAGGCCAATTTG-3’	75.02
	JHA15-pET29b-RcV	5’-AAAAA**CTCGAG**CTGCTGTTTCATAGTTTCCTCAATC-3’	66.96

## Data Availability

All data generated or analyzed during this study are included in this published article and its Supplementary files.

## List of abbreviations

Ae. aegypti: Aedes aegypti
DENV: Dengue virus
ZIKV: Zika virus
CHIKV: Chikungunya virus
E. coli: Escherichia coli
AaLT: *Ae. aegypti* late trypsin
AaET: *Ae. aegypti* early trypsin
AaSPV: *Ae. aegypti* serine protease V
JHA15: juvenile hormone associated 15 protease
AaSPI: *Ae. aegypti* serine protease I
AaCHYMO: *Ae. aegypti* chymotrypsin
IPTG: isopropyl-β-D-thiogalactopyranoside

## References

[1] Ferrer-Miralles N, Saccardo P, Corchero JL, Xu Z, Garcia-Fruitos E. General introduction: recombinant protein production and purification of insoluble proteins. Methods Mol Biol. 2015;1258:1–24. 25447856 10.1007/978-1-4939-2205-5_1

[2] Nguyen JT, Fong J, Fong D, Fong T, Lucero RM, Gallimore JM, Soluble expression of recombinant midgut zymogen (native propeptide) proteases from the Aedes aegypti Mosquito Utilizing E. coli as a host. BMC Biochem. 2018;19(1):12. 30563449 10.1186/s12858-018-0101-0 PMC6299515

[3] Bhatwa A, Wang W, Hassan YI, Abraham N, Li XZ, Zhou T. Challenges Associated With the Formation of Recombinant Protein Inclusion Bodies in Escherichia coli and Strategies to Address Them for Industrial Applications. Front Bioeng Biotechnol. 2021;9:630551. 33644021 10.3389/fbioe.2021.630551 PMC7902521

[4] Eskandari A, Nezhad NG, Leow TC, Rahman MBA, Oslan SN. Essential factors, advanced strategies, challenges, and approaches involved for efficient expression of recombinant proteins in Escherichia coli. Arch Microbiol. 2024;206(4):152. 38472371 10.1007/s00203-024-03871-2

[5] McElwain L, Phair K, Kealey C, Brady D. Current trends in biopharmaceuticals production in Escherichia coli. Biotechnol Lett. 2022;44(8):917–31. 35796852 10.1007/s10529-022-03276-5

[6] Francis DM, Page R. Strategies to optimize protein expression in E. coli. Curr Protoc Protein Sci. 2010;Chapter 5(1):5 24 1–5 9. 10.1002/0471140864.ps0524s61 PMC7162232 20814932

[7] Incir I, Kaplan O. Escherichia coli as a versatile cell factory: Advances and challenges in recombinant protein production. Protein Expr Purif. 2024;219:106463. 38479588 10.1016/j.pep.2024.106463

[8] Mital S, Christie G, Dikicioglu D. Recombinant expression of insoluble enzymes in Escherichia coli: a systematic review of experimental design and its manufacturing implications. Microb Cell Fact. 2021;20(1):208. 34717620 10.1186/s12934-021-01698-w PMC8557517

[9] Ojima-Kato T. Advances in recombinant protein production in microorganisms and functional peptide tags. Biosci Biotechnol Biochem. 2024;89(1):1–10. 39479788 10.1093/bbb/zbae147

[10] Rosano GL, Morales ES, Ceccarelli EA. New tools for recombinant protein production in Escherichia coli: A 5-year update. Protein Sci. 2019;28(8):1412–22. 31219641 10.1002/pro.3668 PMC6635841

[11] Joseph BCP S.; Srimeenakshi S.; Murthy M.; Selvakumar K.; Ganesan M.; Manjunath S. R. An Overview of the Parameters for Recombinant Protein Expression in Escherichia coli. J Cell Sci Ther. 2015;6(5).

[12] Jia B, Jeon CO. High-throughput recombinant protein expression in Escherichia coli: current status and future perspectives. Open Biol. 2016;6(8). 10.1098/rsob.160196 PMC5008019 27581654

[13] O’Donoghue AJ, Liu C, Simington CJ, Montermoso S, Moreno-Galvez E, Serafim MSM, Comprehensive proteolytic profiling of Aedes aegypti mosquito midgut extracts: Unraveling the blood meal protein digestion system. PLoS Negl Trop Dis. 2025;19(2):e0012555. 39913535 10.1371/journal.pntd.0012555 PMC11838913

[14] Ramirez AG, Isoe J, Serafim MSM, Fong D, Le MA, Nguyen JT, Biochemical and physiological characterization of Aedes aegypti midgut chymotrypsin. Sci Rep. 2025;15(1):9685. 40113878 10.1038/s41598-025-93413-7 PMC11926125

[15] Bisia M, Montenegro-Quinonez CA, Dambach P, Deckert A, Horstick O, Kolimenakis A, Secondary vectors of Zika Virus, a systematic review of laboratory vector competence studies. PLoS Negl Trop Dis. 2023;17(8):e0011591. 37651473 10.1371/journal.pntd.0011591 PMC10499269

[16] Tajik S, Farahani AV, Ardekani OS, Seyedi S, Tayebi Z, Kami M, Zika virus tropism and pathogenesis: understanding clinical impacts and transmission dynamics. Virol J. 2024;21(1):271. 39472938 10.1186/s12985-024-02547-z PMC11523830

[17] de Souza WM, Ribeiro GS, de Lima STS, de Jesus R, Moreira FRR, Whittaker C, Chikungunya: a decade of burden in the Americas. Lancet Reg Health Am. 2024;30:100673. 38283942 10.1016/j.lana.2023.100673 PMC10820659

[18] Abbasi E. Aedes aegypti and dengue: insights into transmission dynamics and viral lifecycle. Epidemiol Infect. 2025;153:e88. 40747604 10.1017/S0950268825100320 PMC12345063

[19] Umar K, Sutradhar T, Prakash P, Bavanilatha M, Hemamalani AU, Prakashini RS, Dengue virus: structure, genome, evolution and challenges to control and prevent transmission. Antonie Van Leeuwenhoek. 2025;118(9):139. 40858865 10.1007/s10482-025-02153-1

[20] Lepore L, Vanlerberghe V, Verdonck K, Metelo E, Diallo M, Van Bortel W. Vector control for Aedes aegypti and Aedes albopictus mosquitoes implemented in the field in sub-Saharan Africa: A scoping review. PLoS Negl Trop Dis. 2025;19(7):e0013203. 40632827 10.1371/journal.pntd.0013203 PMC12240363

[21] Martin E, Medeiros MCI, Carbajal E, Valdez E, Juarez JG, Garcia-Luna S, Surveillance of Aedes aegypti indoors and outdoors using Autocidal Gravid Ovitraps in South Texas during local transmission of Zika virus, 2016 to 2018. Acta Trop. 2019;192:129–37. 30763563 10.1016/j.actatropica.2019.02.006

[22] Conway MJ, Haslitt DP, Swarts BM. Targeting Aedes aegypti Metabolism with Next-Generation Insecticides. Viruses. 2023;15(2). 10.3390/v15020469 PMC9964334 36851683

[23] Wang Y, Wang X, Brown DJ, An M, Xue RD, Liu N. Insecticide resistance: Status and potential mechanisms in Aedes aegypti. Pestic Biochem Physiol. 2023;195:105577. 37666603 10.1016/j.pestbp.2023.105577

[24] Tussey DA, Morreale R, Carvalho DO, Stenhouse S, Lloyd AM, Hoel DF, Developing methods for chilling, compacting, and sterilizing adult Aedes aegypti (Diptera: Culicidae) and comparing mating competitiveness between males sterilized as adults versus pupae for sterile male release. J Med Entomol. 2023;60(5):1038–47. 37341187 10.1093/jme/tjad079

[25] Pinto SB, Riback TIS, Sylvestre G, Costa G, Peixoto J, Dias FBS, Effectiveness of Wolbachia-infected mosquito deployments in reducing the incidence of dengue and other Aedes-borne diseases in Niteroi, Brazil: A quasi-experimental study. PLoS Negl Trop Dis. 2021;15(7):e0009556. 34252106 10.1371/journal.pntd.0009556 PMC8297942

[26] Rascon AA Jr., Gearin J, Isoe J, Miesfeld RL. In vitro activation and enzyme kinetic analysis of recombinant midgut serine proteases from the Dengue vector mosquito Aedes aegypti. BMC Biochem. 2011;12:43. 21827688 10.1186/1471-2091-12-43 PMC3162888

[27] Bian G, Raikhel AS, Zhu J. Characterization of a juvenile hormone-regulated chymotrypsin-like serine protease gene in Aedes aegypti mosquito. Insect Biochem Mol Biol. 2008;38(2):190–200. 18207080 10.1016/j.ibmb.2007.10.008 PMC2253661

[28] Isoe J, Rascon AA Jr., Kunz S, Miesfeld RL. Molecular genetic analysis of midgut serine proteases in Aedes aegypti mosquitoes. Insect Biochem Mol Biol. 2009;39(12):903–12. 19883761 10.1016/j.ibmb.2009.10.008 PMC2818436

[29] Lobstein J, Emrich CA, Jeans C, Faulkner M, Riggs P, Berkmen M. SHuffle, a novel Escherichia coli protein expression strain capable of correctly folding disulfide bonded proteins in its cytoplasm. Microb Cell Fact. 2012;11:56. 22569138 10.1186/1475-2859-11-56 PMC3526497

[30] Pouresmaeil M, Azizi-Dargahlou S. Factors involved in heterologous expression of proteins in E. coli host. Arch Microbiol. 2023;205(5):212. 37120438 10.1007/s00203-023-03541-9 PMC10148705

[31] Rizkia PRS S.; Hasan K.; Kamaraa D.S.; Subroto T.; Soemitroa S.; Maksuma I. P. Effect of Isopropyl-β-D-thiogalactopyranoside concentration on prethrombin-2 recombinan gene expression in Escherichia coli ER2566. Procedia Chemistry. 2015;17:118–24.

[32] Oganesyan N, Ankoudinova I, Kim SH, Kim R. Effect of osmotic stress and heat shock in recombinant protein overexpression and crystallization. Protein Expr Purif. 2007;52(2):280–5. 17126029 10.1016/j.pep.2006.09.015 PMC1865119

[33] Ohgane K.; Yoshioka H. Quantification of Gel Bands by an Image J Macro, Band/Peak Quantification Tool 2019 [Available from: https://www.protocols.io/view/quantification-of-gel-bands-by-an-image-j-macro-ba-bp2l6n4bkgqe/v1].

[34] Alonso Villela SM, Kraiem H, Bouhaouala-Zahar B, Bideaux C, Aceves Lara CA, Fillaudeau L. A protocol for recombinant protein quantification by densitometry. Microbiologyopen. 2020;9(6):1175–82. 32255275 10.1002/mbo3.1027 PMC7294310

[35] Rasband WS. ImageJ. National Institutes of Health, Bethesda, Maryland, USA 1997–2015 [Available from: https://imagej.net/ij/].

[36] Zhang ZX, Nong FT, Wang YZ, Yan CX, Gu Y, Song P, Strategies for efficient production of recombinant proteins in Escherichia coli: alleviating the host burden and enhancing protein activity. Microb Cell Fact. 2022;21(1):191. 36109777 10.1186/s12934-022-01917-y PMC9479345

[37] Poureini F, Babaeipour V, Sajedi RH, Khalilzadeh R. Bioprocess Engineering Strategies for the Overproduction of Surface-Expressed Protein in Escherichia coli: A Review. Biotechnol Appl Biochem. 2025. 10.1002/bab.70034 40883223

[38] de Marco A. Recent advances in recombinant production of soluble proteins in E. coli. Microb Cell Fact. 2025;24(1):21. 39815265 10.1186/s12934-025-02646-8 PMC11736966

